# Xylazine in Ohio: Insights from individuals with criminal legal system involvement and a history of illicit stimulant use

**DOI:** 10.1016/j.dadr.2026.100433

**Published:** 2026-03-20

**Authors:** Bridget Duffy, Ashley Short Mejia, Saroj Bista, Gary A. Smith, Nichole L. Michaels

**Affiliations:** a Center for Injury Research and Policy, Abigail Wexner Research Institute at Nationwide Children’s Hospital, 575 Children’s Crossroad, Columbus, OH 43215, USA; b Department of Pediatrics, College of Medicine, The Ohio State University, 370 W. 9th Avenue, Columbus, OH 43210, USA; c College of Public Health, The Ohio State University, 1841 Neil Avenue, Columbus, OH 43210, USA

**Keywords:** Xylazine, Illicit drugs, Illicit stimulants, People who use drugs, Harm reduction, Overdose

## Abstract

**Background:**

Xylazine, a non-opioid sedative not approved for human use, poses serious health risks. It is primarily found in illicitly manufactured fentanyl, but xylazine-fentanyl combinations also appear as illicit stimulant adulterants. Although strategies exist to reduce the risk of xylazine-related harms, some high-risk populations may be unaware of the drug, including people with recent criminal legal system involvement who use illicit stimulants.

**Methods:**

A cross-sectional electronic survey was conducted in Ohio in October 2024 to assess xylazine-related knowledge, attitudes, and behaviors among people with criminal legal system involvement and prior illicit stimulant use. Participants were recruited from Ohio courts.

**Results:**

Among 229 respondents (43.5% response rate), 42.4% had heard of xylazine. The sample was 54.6% female, 83.3% White, 3.1% Hispanic or Latino, and mean age was 38.5 years (SD = 9.4). Individuals who had heard of xylazine were more likely to have used fentanyl (35.1% vs. 7.1%) or heroin (35.5% vs. 2.4%) in the past 6 months than those who had not heard of it. Most respondents (75.1%) indicated it was “very important” to know if xylazine is in their drugs and 66.4% would be “very” or “somewhat likely” to use xylazine test strips, if available at no cost. Among people who had heard of xylazine, 83.5% did not want to use xylazine and try to avoid it.

**Conclusions:**

Most respondents were unaware of xylazine, despite its prevalence in Ohio. Xylazine education and easy access to harm reduction tools are critical for preventing xylazine-related harms among this high-risk population.

## Highlights

• Ohioans who use illicit stimulants and have criminal legal system involvement were surveyed about xylazine.
• Most survey respondents had not heard of xylazine.
• Among people with prior knowledge of the drug, 8 in 10 said they do not want to use xylazine and try to avoid it.
• The majority of respondents indicated they would use xylazine test strips, if available at no cost.

## 1 Introduction

Xylazine is a non-opioid sedative and tranquilizer with analgesic and muscle relaxant effects approved by the United States (US) Food and Drug Administration for use in veterinary medicine (Drug Enforcement Administration, 2022, National Institute on Drug Abuse, 2024). Although not approved for human use, xylazine has been identified as an adulterant in the US illicit drug supply, posing significant and unique threats to human health. Symptoms of xylazine consumption include sedation, slowed heart rate, difficulty breathing, low blood pressure, and severe skin wounds that can become infected and difficult to treat (Centers for Disease Control and Prevention, 2024). Xylazine was first reported in the US illicit drug supply in Puerto Rico in 2001 but has since been identified in nearly all states (Torruella, 2011, Gupta et al., 2023, Wong et al., 2008; Drug Enforcement Administration, n.d.). In the US, xylazine is rarely detected in overdose deaths without illegally manufactured fentanyl also being present (Hays et al., 2024, Kariisa et al., 2023, Spencer et al., 2023). Since 2021, a low but growing prevalence of xylazine in stimulants has been reported, rarely without fentanyl, although it is unclear if this reflects current polysubstance use patterns or exposure driven by adulteration of the drug supply (Bradford et al., 2024, Delcher et al., 2023, Friedman et al., 2022, Martin et al., 2025, StreetCheck, 2026, Thangada et al., 2021, Wagner et al., 2023).

In 2019, there were 15 unintentional overdose deaths involving xylazine in Ohio, a number that has increased significantly to 373 deaths in 2024 (Ohio Department of Health, 2024, Ohio Department of Health, 2025). As documented elsewhere in the US, nearly all xylazine-involved deaths in Ohio also involved fentanyl or other opioids (Ohio Department of Health, 2025). Ohio had one of the highest rates of xylazine detected in drug seizure samples from US states between 2019 and 2022 (Cano et al., 2024). An analysis of 19,529 crime lab samples across 87 Ohio counties from April through December 2023 found 10.5% were positive for xylazine, and among those, 96.8% also contained fentanyl, 22.0% cocaine, and 12.4% methamphetamine (Rosenblum et al., 2025). In March 2023, Ohio Governor Mike DeWine implemented an emergency executive order classifying xylazine as a Schedule III controlled substance, but this change has not resulted in an observable decrease in xylazine-involved overdose deaths (Ohio Department of Health, 2025, State of Ohio Office of the Governor, 2023).

A recent study exploring xylazine awareness and attitudes among Ohioans reporting use of any illicit drugs within the past 6 months found that more than half of respondents were unaware of xylazine, the majority who were aware of it did not want to use it, and most felt it was important to know if xylazine was in their drugs (Michaels et al., 2024). Additional research has documented mixed responses to xylazine awareness and perceived risk, with some people who use drugs increasing harm reduction behaviors or indicating a willingness to use tools such as xylazine test strips, while others report no behavior change or a preference for xylazine over other drugs (Kelly et al., 2025, Salwan et al., 2025, Shrestha et al., 2025, Sibley et al., 2025).

Substance use is common among individuals with criminal legal system involvement, with one nationally representative US study finding that among people with criminal legal system involvement within the previous year, more than half had a substance use disorder (Shah et al., 2024). Individuals who return to drug use following community reentry are at increased risk of overdose and other drug-related harms due to decreased drug tolerance and lack of knowledge regarding the current drug supply, including emerging adulterants such as xylazine (Boulger et al., 2022).

Polysubstance use is also frequently reported among individuals with a history of criminal legal system involvement (Ford et al., 2022). People who use illicit stimulants are a particularly important population for xylazine-related research. Because xylazine is nearly always paired with fentanyl, with rare or sporadic presence in stimulants alone, individuals who primarily use illicit stimulants may be less aware of the drug. As a result, this population may be less likely than individuals who use opioids to anticipate xylazine in their drugs or recognize xylazine-related symptoms, thereby increasing their vulnerability to negative effects of the drug. Given the current low prevalence of xylazine in stimulant supplies, awareness of its risks and appropriate harm reduction strategies may help individuals who use stimulants avoid exposure, particularly in the context of polysubstance use and an evolving drug supply.

Xylazine remains a largely unexamined public health concern for people who use illicit stimulants, supporting the need for additional research with populations at high risk for exposure and related harms, including individuals with recent criminal legal system involvement. This cross-sectional study describes xylazine knowledge, attitudes, and behaviors among Ohioans involved in the criminal legal system who have a self-reported history of illicit stimulant use. Given that xylazine awareness may be lower among people who use illicit stimulants, compared to those who primarily use opioids, we also describe differences in xylazine awareness by types of drugs used.

## 2 Methods

### 2.1 Survey development and study population

The study team developed an electronic survey in Research Electronic Data Capture (REDCap) to gauge xylazine-related knowledge, attitudes, and behaviors among court-involved individuals in Ohio with a self-reported history of illicit stimulant use. Survey development involved sharing a draft created by the study team with community partners and incorporating their feedback prior to its implementation. Twelve people who were not part of the study team were involved in this process, including 2 people who disclosed lived experience with substance use, public health, behavioral health, and harm reduction professionals, physicians, researchers, and community service providers. All had experience working in the field of drug use and substance use disorders in their respective professional roles.

This one-time, cross-sectional supplemental survey collected responses from individuals who were enrolled in an ongoing cluster randomized trial investigating fentanyl test strips as a harm reduction strategy for preventing overdoses. The study enrolled eligible individuals from 27 participating court sites in 24 counties throughout Ohio beginning in November 2022. Participant recruitment in the randomized trial was conducted in-person at court sites and was ongoing at the time of the current study, but has since closed. Participant eligibility criteria included: ≥ 18 years, able to complete study activities in English, access to a phone or email (necessary for follow up in the randomized trial), and self-reported illicit stimulant use (i.e., cocaine/crack cocaine, methamphetamine/amphetamine) or polysubstance use including illicit stimulants within the past 12 months. Potential participants were screened with the question, “Have you used any illicit stimulants in the past year?” Eligible individuals also had to be involved with the court recruitment site as a participant in a specialized docket (i.e., drug court), a probation/community supervision agreement, or other matter pending before the court. Prior to data collection, all participants provided informed consent. For this study, the REDCap survey links were sent to all active participants via text or email, according to participant preferences. Sociodemographic data was collected at the time of enrollment in the randomized trial and was not repeated for the current study. This study was approved as a modification to the randomized trial study by the Institutional Review Board at the authors’ institution.

### 2.2 Data collection

Participants enrolled in the randomized trial were informed that a one-time optional survey would be available for them to participate in for a limited time. A link to the xylazine survey in REDCap was electronically distributed to 527 individuals on October 21, 2024, via participants’ preferred contact method (text or email). We used the Automated Survey Invitations feature in REDCap, which generates unique survey links that allow only one response per participant. On the first page of the electronic survey, participants were provided with additional information about the study’s confidentiality and privacy procedures to review before proceeding. Individuals who completed the survey received $25 on the ClinCard they were issued as part of the randomized trial and new payment cards were issued to participants as needed. The survey was closed after 24 h because we reached our goal of at least 220 responses. This limit was determined based on budgetary constraints.

#### 2.2.1 Measures

##### 2.2.1.1 Baseline sociodemographic data gathered as part of the randomized trial

Age was recorded in years. Race was categorized as White, Black, two or more races, other, or missing/unknown. Ethnicity was classified as Hispanic or Latino or not Hispanic or Latino. Education level was categorized as did not finish high school, finished high school, or additional schooling beyond high school. Employment status was recorded as currently had a job (yes/no), and homelessness was assessed as currently experiencing homelessness (yes/no).

##### 2.2.1.2 Xylazine and drug use

Survey questions focused on drug use, xylazine awareness and knowledge, and pilot questions regarding awareness of two emerging drugs (nitazenes and tianeptine). Responses were stratified by whether the participant had ever heard of xylazine (yes/no). The question was asked as follows: “Now, we'd like to ask you about a specific drug. Have you ever heard of xylazine (pronounced: ZY-luh-zeen) (also called "tranq," "tranq dope," "Philly dope," "sleep cut," or "zombie drug")?” Individuals who indicated “yes” were then directed to an open response question asking what they knew about xylazine. Next, participants were asked if they had used recreational or “street” drugs in the past 6 months (yes/no). If they selected “yes,” branching logic revealed several more questions about their drug use in the past 6 months, including what drugs they used (select all that apply), what methods of drug use they used (select all that apply), their average frequency of drug use, whether they typically used drugs alone or with other people, and whether they experienced an overdose.

##### 2.2.1.3 Xylazine description

A description of xylazine was then provided before further questions were asked. This information was not visible to participants prior to this point in the survey. The description provided was: “Xylazine is a drug used by veterinarians to relax or make animals sleepy. It is not approved for human use. When people use xylazine, it can make them very sleepy or not able to be woken up. It can lower their blood pressure, slow their heart rate, and decrease their breathing. It can also cause serious skin wounds, even in people who do not inject the drug. Xylazine is often found in drugs that also contain fentanyl.” After this description was provided, individuals who indicated recreational or “street” drug use within the past 6 months were asked about the likelihood that they had used drugs containing xylazine during this time. Then, all study participants (regardless of drug use within the past 6 months) were asked how important it is to know if xylazine is in their drugs and how likely they would be to use xylazine test strips if they were available at no cost.

##### 2.2.1.4 Xylazine only

Several additional questions were presented only to participants who reported that they had heard of xylazine prior to the survey. These individuals were asked how they first heard of xylazine, their attitude about using xylazine, and whether they had ever used xylazine test strips to test their drugs before using them. This group of participants was also provided with an open response question asking for more information about why they feel the way they do about xylazine.

##### 2.2.1.5 Other emerging drugs

To gauge their awareness of emerging drugs in Ohio, all participants were asked two additional questions about whether they had ever heard of nitazenes or tianeptine.

### 2.3 Data analysis

Sociodemographic and survey measures were compared between individuals who reported prior awareness of xylazine and those who had not heard of the drug using chi-square and Fisher’s exact tests; a *t*-test was used to compare mean ages between these groups. P-values < .05 indicated statistical significance. All quantitative analyses were conducted in RStudio (version 2022.02.3) using the dplyr and lubridate packages for data manipulation and processing (Grolemund and Wickham, 2011, Wickham et al., 2023). The brief answers that participants provided to the two open response questions were systematically categorized using an inductive qualitative content analysis approach. Codebooks were developed by the study team for each question and codes were applied by the first author using Microsoft Excel to organize the data. Multiple codes could be applied to each response. A second coder from the study team independently coded the data to ensure reliability. Discrepancies were discussed and resolved through consensus.

## 3 Results

### 3.1 Characteristics of the overall sample

A total of 229 individuals (229/527, 43.5% response rate) completed the survey. The mean age of respondents was 38.5 years, 54.6% identified as female, 83.3% were White, and 3.1% were Hispanic or Latino (Table 1). Most individuals had finished high school (41.5%) or additional schooling beyond high school (39.7%). Many did not have a job (59.0%) and 13.6% were currently experiencing homelessness.

**Table 1:** Sociodemographic and drug use characteristics by prior awareness of xylazine (N = 229).

**Characteristic**	**n (%)**^a^			
	**Overall**	**Ever heard of xylazine**		
		**Yes**	**No**	p-value*
**Total**	**229 (100.0)**	**97 (42.4)**	**132 (57.6)**	
**Age** (mean, sd)	38.5, 9.4	36.5, 7.9	39.9, 10.1	**0.005**
**Sex**				0.058
Male	104 (45.4)	37 (38.1)	67 (50.8)	
Female	125 (54.6)	60 (61.9)	65 (49.2)	
**Race**				0.302
White	189 (83.3)	82 (85.4)	107 (81.7)	
Black	25 (11.0)	7 (7.3)	18 (13.7)	
Two or more races	6 (2.6)	4 (4.2)	2 (1.5)	
Other^b^	7 (3.1)	3 (3.1)	4 (3.1)	
Missing/Unknown^c^	2	1	1	
**Ethnicity**				1.000
Hispanic or Latino	7 (3.1)	3 (3.1)	4 (3.0)	
Not Hispanic or Latino	222 (96.9)	94 (96.9)	128 (97.0)	
**Education**				0.344
Did not finish high school	43 (18.8)	19 (19.6)	24 (18.2)	
Finished high school	95 (41.5)	35 (36.1)	60 (45.5)	
Additional schooling beyond high school	91 (39.7)	43 (44.3)	48 (36.4)	
**Current job**				0.748
Yes	94 (41.1)	41 (42.3)	53 (40.2)	
No	135 (59.0)	56 (57.7)	79 (59.8)	
**Currently experiencing homelessness**				0.751
Yes	31 (13.6)	14 (14.4)	17 (13.0)	
No	197 (86.4)	83 (85.6)	114 (87.0)	
Missing/Unknown^c^	1	0	1	
**In the past 6 months, have you used any recreational or "street" drugs?**				0.982
Yes	73 (31.9)	31 (32.0)	42 (31.8)	
No	156 (68.1)	66 (68.0)	90 (68.2)	
**In the past 6 months, what drugs have you used?**^d^, ^e^				
Any illicit stimulant^f^	48 (65.8)	23 (74.2)	25 (59.5)	0.192
Cocaine, crack	29 (39.7)	16 (51.6)	13 (31.0)	0.075
Methamphetamine	31 (42.5)	16 (51.6)	15 (35.7)	0.174
Marijuana	43 (58.9)	17 (54.8)	26 (61.9)	0.544
Fentanyl^g^	14 (19.2)	11 (35.5)	3 (7.1)	**0.005**
Heroin	12 (16.4)	11 (35.5)	1 (2.4)	**< 0.001**
Prescription medication^h^	13 (17.8)	6 (19.4)	7 (16.7)	0.767
Other^i^	12 (16.4)	7 (22.6)	5 (11.9)	0.224
**In the past 6 months, how have you used drugs?**^d^, ^e^				
Injecting	15 (20.6)	11 (35.5)	4 (9.5)	**0.009**
Smoking	61 (83.6)	24 (77.4)	37 (88.1)	0.224
Snorting	31 (42.5)	19 (61.3)	12 (28.6)	**0.005**
By mouth	20 (27.4)	12 (38.7)	8 (19.1)	0.063
**On average, how often have you used drugs in the past 6 months?**^e^				0.887
Less than once a day	33 (45.2)	15 (48.4)	18 (42.9)	
Once a day	12 (16.4)	5 (16.1)	7 (16.7)	
More than once a day	28 (38.4)	11 (35.5)	17 (40.5)	
**When you used drugs in the past 6 months, did you usually use alone or with other people?**^e^				0.079
Alone	37 (50.7)	12 (38.7)	25 (59.5)	
With other people	36 (49.3)	19 (61.3)	17 (40.5)	
**Have you had an overdose in the past 6 months?**^e^				**0.045**
Yes	4 (5.5)	4 (12.9)	0 (0.0)	
No	65 (89.0)	25 (80.7)	40 (95.2)	
Not sure	4 (5.5)	2 (6.5)	2 (4.8)	
**In the past 6 months, how likely is it that you have used drugs containing xylazine?**^e^				**< 0.001**
Very likely	7 (9.6)	7 (22.6)	0 (0.0)	
Somewhat likely	5 (6.9)	4 (12.9)	1 (2.4)	
Not likely or unlikely	11 (15.1)	3 (9.7)	8 (19.1)	
Somewhat unlikely	5 (6.9)	3 (9.7)	2 (4.8)	
Very unlikely	45 (61.6)	14 (45.2)	31 (73.8)	
**How important is it to know if xylazine is in your drugs?**				0.151
Very important	172 (75.1)	70 (72.2)	102 (77.3)	
Somewhat important	17 (7.4)	5 (5.2)	12 (9.1)	
Not important or unimportant	20 (8.7)	11 (11.3)	9 (6.8)	
Somewhat unimportant	3 (1.3)	3 (3.1)	0 (0.0)	
Very unimportant	17 (7.4)	8 (8.3)	9 (6.8)	
**How likely would you be to use xylazine test strips if they were available to you at no cost?**				0.549
Very likely	119 (52.0)	51 (52.6)	68 (51.5)	
Somewhat likely	33 (14.4)	14 (14.4)	19 (14.4)	
Not likely or unlikely	30 (13.1)	14 (14.4)	16 (12.1)	
Somewhat unlikely	8 (3.5)	1 (1.0)	7 (5.3)	
Very unlikely	39 (17.0)	17 (17.5)	22 (16.7)	
**Have you ever heard of drugs called nitazenes or "ISO" or "zenes" or "Frankenstein"?**				**0.001**
Yes	15 (6.6)	13 (13.4)	2 (1.5)	
No	201 (87.8)	78 (80.4)	123 (93.2)	
Not sure	13 (5.7)	6 (6.2)	7 (5.3)	
**Have you ever heard of a drug called tianeptine, also called "gas station heroin" or "Tianna Red" or "Zaza"?**				0.105
Yes	20 (8.7)	12 (12.4)	8 (6.1)	
No	193 (84.3)	76 (78.4)	117 (88.6)	
Not sure	16 (7.0)	9 (9.3)	7 (5.3)	

⁎ Bold indicates statistical significance. Tests include *t*-test, chi-square, Fisher’s exact.

a Column percentage may not sum to 100.0% due to rounding error.

b Includes Native American or Alaska Native and open responses entered by the respondent (e.g., “Mexican”).

c Missing/Unknown values were omitted from the denominator when calculating percentages.

d Not mutually exclusive.

e Denominator is individuals who answered yes to: “In the past 6 months, have you used any recreational or "street" drugs?”

f Includes cocaine, crack, and methamphetamine.

g Includes fentanyl and fentanyl analogs.

h Prescription medication includes prescription pain medicine (e.g., oxycodone, Percocet), sedatives (e.g., benzodiazepines, Xanax) or muscle relaxers, and stimulants (e.g., Adderall). Participants were asked to include only drugs that were not used as directed by a doctor.

i Includes hallucinogens, inhalants, k2/spice, ketamine, MDMA, xylazine, and open responses.

### 3.2 Xylazine and drug use characteristics

Among respondents, 42.4% had ever heard of xylazine, while 57.6% had not. Table 1 compares sociodemographic and drug use characteristics between these groups. No statistically significant sociodemographic differences were observed between the two xylazine awareness (yes/no) groups, except for age. Individuals who had heard of xylazine were younger, on average, (mean age: 36.5 years vs. 39.9 years, p = .005) than those who had not.

Similar proportions of both groups reported having used recreational or “street” drugs in the past 6 months. Among respondents who reported having used illicit drugs in the past 6 months, 55.2% (16/29) of people who used cocaine and 51.6% (16/31) of people who used methamphetamine had heard of xylazine, compared to 78.6% (11/14) of people who used fentanyl. Individuals who had heard of xylazine reported having used cocaine/crack (51.6%) or methamphetamine (51.6%) more often than those who had not heard of xylazine (31.0% and 35.7%, respectively), but these differences were not statistically significant. Significantly more people who had heard of xylazine had used fentanyl (35.5% vs. 7.1%; p = .005) or heroin (35.5% vs. 2.4%; p < .001) in the past 6 months, compared to those who had not heard of xylazine.

Greater proportions of people who had heard of xylazine used drugs by injecting or snorting in the past 6 months, compared to those who had not heard of xylazine (35.5% vs. 9.5%; p = .009 and 61.3% vs. 28.6%; p = .005, respectively). The majority of people who had heard of xylazine indicated that they usually used drugs with other people, rather than alone (61.3%). In contrast, most people who had not heard of xylazine reported that they usually used drugs alone (59.5%), however these differences were not statistically significant. All individuals (n = 4, 5.5%) who reported personally experiencing an overdose in the past 6 months had heard of xylazine.

### 3.3 Measures gathered after providing all respondents with a description of xylazine

Among respondents who reported having used illicit drugs in the past 6 months, greater proportions of individuals who had heard of xylazine reported it being “very likely” (22.6%) or “somewhat likely” (12.9%) that they had used drugs containing xylazine during that time, compared to people who had not heard of xylazine (0.0% and 2.4%, respectively; p < .001).

Among all study participants, including those who did not report illicit drug use within the past 6 months, the majority (75.1%) indicated it was “very important” to know if xylazine is in their drugs, with no significant differences between respondents with and without prior knowledge of xylazine. Likewise, similar proportions of both groups (52.6% and 51.5%) reported they would be “very likely” to use xylazine test strips if they were available to them at no cost.

### 3.4 Emerging drugs

Among all survey respondents, most had not heard of nitazenes (87.8%) or tianeptine (84.3%) (Table 1). A greater proportion of people who had heard of xylazine had also heard of nitazenes, compared to those who had not heard of xylazine (13.4% vs. 1.5%; p = .001). Similarly, more individuals who had heard of xylazine reported ever having heard of tianeptine compared to those who had not heard of xylazine, although this difference was not significant (12.4% vs. 6.1%; p = .105).

### 3.5 Xylazine specific characteristics

Several questions were asked only of individuals who reported having heard of xylazine prior to taking the survey. When asked the open response question, “Briefly, what do you know about xylazine?” many participants mentioned the potential harm associated with xylazine use, particularly that it was dangerous or unsafe (Table 2). Nearly one-quarter of respondents specifically mentioned the risk of death and a similar percentage noted concerns about the behavioral or psychological effects of xylazine. Some individuals identified that naloxone does not reverse the effects of xylazine (15.5%).

**Table 2:** Coded open responses to the question “Briefly, what do you know about xylazine?” among individuals with prior awareness of the drug (n = 97).

**Code**^a^	**Definition**	**n (%)**	**Illustrative Responses**
Potential harm	Perceived risk or harmful/negative consequences or unwanted effects (without specifying death)	27 (27.8)	*“It is very dangerous.”* *“It’s extremely unsafe.”*
Fatal risk	Xylazine can cause death	24 (24.7)	*“That it’s really strong and it kills you.”* “*It’s deadly, the most dangerous drug out right now.”* *“It’s a quick killer.”* *“Dead body drug.”*
Behavioral/psychological effects	Xylazine causes behavioral/psychological alterations (e.g., sedation)	23 (23.7)	“*I know that it’s a tranquilizer and I seen people that have used it and they act different while on it*.” “*It will make you disoriented*.”
Naloxone	Naloxone (i.e., Narcan) does not reverse the effects of xylazine	15 (15.5)	“*Narcan won’t help*.” “*Overdoses from it are not* *reversed with Narcan*.”
Strong effects	Powerful or potent effect of xylazine, including heightening the effects of other drugs like fentanyl.	13 (13.4)	“*It’s very very potent*.” “*It makes fentanyl stronger*.”
Cutting agent	Xylazine is used to cut other drugs or is mixed with other drugs.	13 (13.4)	“*It’s used as a cut or added into fentanyl*.” “*Often mixed with fentanyl and other drugs*.” “*It’s what they mix in to make you fall asleep*.”
Lack of knowledge	Expression of lacking knowledge about xylazine	10 (10.3)	“*Nothing really.”* “*Not much but the name*.”
Physical health effects	Xylazine causes physical health changes.	7 (7.2)	“*Causes wounds that won’t heal*.” “*That it eats the body.”*

a Codes are not mutually exclusive.

When asked how they first heard of xylazine, the most common responses were from a friend (21.7%), health care provider (13.4%), or the internet (13.4%) (Table 3). When asked “How do you feel about using xylazine?” the majority of individuals with prior knowledge of the drug reported they did not want to use xylazine and tried to avoid it (83.5%). Survey participants were then given the open response question: “Why do you feel that way about xylazine?” Table 4 displays the reasons provided by individuals who indicated that they do not want to use xylazine and try to avoid it. A large proportion (44.4%) of respondents cited the physical or mental effects of xylazine, such as concerns about its negative effects on the skin and the potential for losing consciousness while using the drug.

**Table 3:** Xylazine-related knowledge, attitudes, and behaviors among respondents with prior awareness of xylazine (n = 97).

**Characteristic**	**n (%)**^a^
**Total**	**97**
**How did you first hear about xylazine?**	
Friend	21 (21.7)
Health care provider	13 (13.4)
Internet	13 (13.4)
My drug supplier/dealer	11 (11.3)
News	11 (11.3)
Peer recovery supporter	7 (7.2)
Court personnel	4 (4.1)
Family member	3 (3.1)
Harm reduction program	1 (1.0)
Other^b^	6 (6.2)
Not sure	7 (7.2)
**How do you feel about using xylazine?**	
I would rather use xylazine than other drugs	3 (3.1)
I use xylazine when it is available, but it isn't my first choice	1 (1.0)
I don’t try to use xylazine, but I'm not worried about it being in my drugs	3 (3.1)
I do not want to use xylazine and try to avoid it	81 (83.5)
Other^c^	9 (9.3)
**Have you ever used xylazine test strips to test your drugs before you used them?**	
Yes	3 (3.1)
No	93 (95.9)
Not sure	1 (1.0)

a Column percentage may not sum to 100.0% due to rounding error.

b Includes other open responses (e.g., client, “just random talk on the street”).

c Includes other open responses (e.g., “it scares me,” “I hate that it exist,” “I would never use it”).

**Table 4:** Coded open responses to “Why do you feel that way about xylazine?” among individuals with prior awareness of the drug who indicated they do not want to use the drug and try to avoid it (n = 81).

**Code**^a^	**Definition**	**n (%)**	**Illustrative Responses**
Effects and perception	Personal, observed, or anticipated physical or mental effects of xylazine—both positive and negative. This includes direct experiences, secondhand reports, and general perceptions, including fears. Excludes explicit mentions of death, which is captured separately.	36 (44.4)	“*I heard it eats your skin and makes you* crazy and take you mentally to another place.” *“Dope with it in it doesn't feel right, causes black out which are dangerous and lead to trouble*.” *“I am afraid of it*.” “*It’s not a good feeling.”*
No longer using drugs	Indication of not using drugs, maintaining sobriety	25 (30.9)	“I *am a addict in recovery*.” “*I no longer want drugs*.”
Fatal risk	Explicit fear of dying from xylazine use or stating that it is deadly.	23 (28.4)	“*I don’t want to die*.” “*It’s deadly*.”
Comparison to other drugs	Comparing xylazine to other drugs (e.g., feeling that xylazine is more harmful than another drug)	5 (6.2)	“*I heard it’s more dangerous than fentanyl*.” “*From what I’ve seen it’s worse than fentanyl*.” “*It’s worse than the K2, spice and bath salts wave was*.”
Limited knowledge	Limited knowledge of xylazine as a deterrent for using it.	5 (6.2)	“*I never know what’s really in it.”* “*Lack of some knowledge.”* “*Because I don’t know what it is*.”
Composition	Concern regarding the composition of xylazine	3 (3.7)	“…*they are putting too much extra shit in that stuff now a days*.” “*Because it’s not made for humans*.”

a Codes are not mutually exclusive.

A very small subgroup of individuals responded to “How do you feel about using xylazine?” by indicating that they do not try to use it, but they are also not worried about it (n = 3), they use it when it is available, but it isn’t their first choice (n = 1), or they would rather use xylazine than other drugs (n = 3) (Table 3). When these individuals were then asked the open response question “Why do you feel that way about xylazine?” several mentioned their need to manage withdrawal symptoms as taking priority over worrying about what might be in the illicit drug supply (e.g. “*[because] if I was in active addiction if sick enough I’m going to do it.”)* Additionally, one respondent who would rather use xylazine than other drugs, stated: “*I have used fentanyl for so many years that it is basically maintenance. I don’t get high. I get high on xylazine*.”

## 4 Discussion

This study surveyed individuals in a Midwestern US state who had criminal legal system involvement and current or past illicit stimulant use to assess their xylazine-related knowledge, attitudes, and behaviors. More than half of respondents had not previously heard of xylazine. This finding is consistent with some recent studies of xylazine awareness in Ohio and elsewhere in the US (Hochheimer et al., 2024, Michaels et al., 2024). However, it contrasts with other studies that reported greater awareness of xylazine in the illicit drug supply (Kelly et al., 2025, Park et al., 2024, Quijano et al., 2023, Shrestha et al., 2025). Although differing levels of xylazine awareness across geographic locations and drug markets are not surprising, it is notable that our study participants reported the least awareness of xylazine. This may be attributed to the fact that our study was conducted in the Midwest, while most prior research on this topic is from the Northeastern region of the US. However, differences in sampling frame between our study and others should also be considered. Our sample consisted of court-involved individuals with a self-reported history of illicit stimulant use. Most other research has focused on broader community samples of people who use drugs (Hochheimer et al., 2024, Kelly et al., 2025, Michaels et al., 2024, Park et al., 2024, Quijano et al., 2023, Shrestha et al., 2025). Also, court-linked supervision programs often maintain abstinence-focused structures. This approach by the courts, along with a sample of people primarily using stimulants, may have limited participants' knowledge of emerging drugs like xylazine.

In the current study, xylazine knowledge among participants was limited. As the illicit drug supply continues to evolve rapidly, people face greater risks of exposure to unidentified and unanticipated adulterants (Friedman et al., 2022, Thangada et al., 2021, Di Trana and Montanari, 2022, Wagner et al., 2023). This lack of awareness may also impede the practice of harm reduction strategies. In a qualitative study of individuals with recent overdose reversal experiences, awareness of xylazine and its risks prompted meaningful changes in drug use behaviors. These included reducing the amount or frequency of use, modifying use practices, or choosing to abstain or seek treatment (Sibley et al., 2025). This suggests that providing education on xylazine and its risks may promote the adoption of harm reduction strategies and represent an opportunity for effective intervention.

Among our study participants who were aware of xylazine prior to the survey, many identified perceived harms and dangers associated with xylazine use, including sedative effects and death. After all participants were provided with a brief description of xylazine and its potential effects on humans, most said it was “very important” to know if xylazine was in their drugs, and the majority indicated they would be “very likely” or “somewhat likely” to use xylazine test strips if they were available at no cost. This is consistent with previous research showing a high level of interest in xylazine test strips among people who use drugs (Quijano et al., 2023, Reed et al., 2022, Shrestha et al., 2025).

Until recently, xylazine test strips were considered drug paraphernalia in Ohio. On October 1, 2025, Ohio Governor Mike DeWine signed an executive order allowing the adoption of a rule decriminalizing test strips and reagent kits for detecting xylazine and certain other drugs (State of Ohio Office of the Governor, 2025). Given that most respondents in our study indicated they would use xylazine test strips, this change in policy creates meaningful opportunities for harm reduction programs to incorporate xylazine test strips into their service delivery, providing people who use drugs a legal way to identify xylazine in their drugs. The impact this legal change has on health and safety will depend on implementation factors such as efforts to increase awareness of xylazine test strips, outreach to populations at greatest risk, and test strip availability and cost. While xylazine test strips reliably detect xylazine at low concentrations, their concentration-dependent false positives with common drugs and adulterants such as lidocaine, methamphetamine, and diphenhydramine, highlight the need for cautious interpretation, underscoring their role as a complementary tool within a broader harm reduction strategy (Scott et al., 2025, Sisco et al., 2024).

It is unsurprising that many individuals in this sample reported no drug use in the past 6 months given that abstinence is a common requirement for court participation or community supervision. For individuals who return to drug use following criminal legal system involvement, lack of awareness regarding changes in the drug supply, combined with reduced drug tolerance, can increase risk of overdose and other harms, including those associated with adulterants like xylazine (Boulger et al., 2022). Ensuring that people with substance use histories who are involved in the criminal legal system receive accurate and up-to-date information about the evolving illicit drug supply, regardless of their current use status, is a critical component of harm reduction for this population.

### 4.1 Limitations

Despite its strengths, there are limitations to consider for this study. Participants were from a specific population living in one Midwestern state, potentially limiting the generalizability of findings. All participants reported past-year stimulant use at enrollment in the parent study. However, a substantial proportion of the analytic sample reported no stimulant or other drug use in the six months preceding this survey administration. This discrepancy likely reflects, at least in part, the timing of data collection, fielded a median of 10 months (range: 0–24 months) after screening/enrollment, as well as abstinence requirements commonly imposed as conditions of court participation or community supervision. We acknowledge this temporal gap and the potential influence of mandated abstinence as important study limitations. Notwithstanding these considerations, among participants who reported drug use in the past six months, nearly two-thirds reported illicit stimulant use (i.e., cocaine, crack cocaine, or methamphetamine). Moreover, stimulant use represented the most prevalent drug class reported in the sample. These findings support the characterization of this population as predominantly composed of individuals who use stimulants. Because the study was cross-sectional, changes in awareness and knowledge of xylazine over time could not be assessed. Survey responses were self-reported by participants, thus there is the potential for social desirability bias and recall bias. The survey used to collect these data is not a validated instrument, however, it was developed with input from two individuals who disclosed lived experience with substance use. Despite limitations, self-reported survey research is a valuable tool for understanding illicit drug use patterns and preferences (Bharat et al., 2023). Our findings contribute important information about xylazine-related knowledge, attitudes, and behaviors among a high-risk population in a Midwestern state.

## 5 Conclusions

Xylazine is an increasingly prevalent adulterant in the illicit drug supply in the US and poses unique personal and public health risks. This study of individuals who are involved in the criminal legal system and have a history of illicit stimulant use found that most participants had not heard of xylazine, representing one of the lowest levels of awareness reported in the literature to date. Despite their initially limited awareness, many respondents, after receiving a brief educational overview of xylazine, shared the belief that it is important to know if xylazine is in their drugs, and more than half indicated they would use xylazine test strips if available at no cost. Most participants who had heard of xylazine prior to taking the survey noted that they did not want to use xylazine and try to avoid it. Although the prevalence of xylazine in stimulants remains relatively low in the US illicit drug market, its continued presence, along with the pervasiveness of polysubstance use and drug adulteration, suggests that xylazine will likely remain an important public health concern for the foreseeable future. Educating people who use drugs about xylazine, including people who use stimulants and may not recognize its risks as well as individuals with recent criminal legal system involvement who may be less aware of current drug supply trends, is critical, as is providing easy access to xylazine test strips and other harm reduction tools.

## CRediT authorship contribution statement

**Ashley Short Mejia:** Writing – review & editing, Project administration. **Bridget Duffy:** Writing – original draft, Project administration, Investigation, Formal analysis, Conceptualization. **Michaels Nichole L:** Writing – review & editing, Supervision, Resources, Funding acquisition, Conceptualization. **Gary A. Smith:** Writing – review & editing, Funding acquisition, Conceptualization. **Saroj Bista:** Writing – review & editing.

## Funding source

This publication was supported in part with a grant (R01CE003349) from the National Center for Injury Prevention and Control at the Centers for Disease Control and Prevention of the U.S. Department of Health and Human Services (10.13039/100000016HHS). The contents are those of the author(s) and do not necessarily represent the official views of, nor an endorsement by, CDC/HHS, or the U.S. Government.

## Declaration of Competing Interest

The authors declare that they have no known competing financial interests or personal relationships that could have appeared to influence the work reported in this paper.
